# Strand asymmetries across genomic processes

**DOI:** 10.1016/j.csbj.2023.03.007

**Published:** 2023-03-11

**Authors:** Camille Moeckel, Apostolos Zaravinos, Ilias Georgakopoulos-Soares

**Affiliations:** a Institute for Personalized Medicine, Department of Biochemistry and Molecular Biology, The Pennsylvania State University College of Medicine, Hershey, PA, USA; b Department of Life Sciences, European University Cyprus, Diogenis Str., 6, Nicosia 2404, Cyprus; c Cancer Genetics, Genomics and Systems Biology laboratory, Basic and Translational Cancer Research Center (BTCRC), Nicosia 1516, Cyprus

**Keywords:** Mutational strand asymmetries, Transcriptional strand asymmetries, Replicative strand asymmetries, Orientation

## Abstract

Across biological systems, a number of genomic processes, including transcription, replication, DNA repair, and transcription factor binding, display intrinsic directionalities. These directionalities are reflected in the asymmetric distribution of nucleotides, motifs, genes, transposon integration sites, and other functional elements across the two complementary strands. Strand asymmetries, including GC skews and mutational biases, have shaped the nucleotide composition of diverse organisms. The investigation of strand asymmetries often serves as a method to understand underlying biological mechanisms, including protein binding preferences, transcription factor interactions, retrotransposition, DNA damage and repair preferences, transcription-replication collisions, and mutagenesis mechanisms. Research into this subject also enables the identification of functional genomic sites, such as replication origins and transcription start sites. Improvements in our ability to detect and quantify DNA strand asymmetries will provide insights into diverse functionalities of the genome, the contribution of different mutational mechanisms in germline and somatic mutagenesis, and our knowledge of genome instability and evolution, which all have significant clinical implications in human disease, including cancer. In this review, we describe key developments that have been made across the field of genomic strand asymmetries, as well as the discovery of associated mechanisms.

## Graphical Abstract

**Figure:**
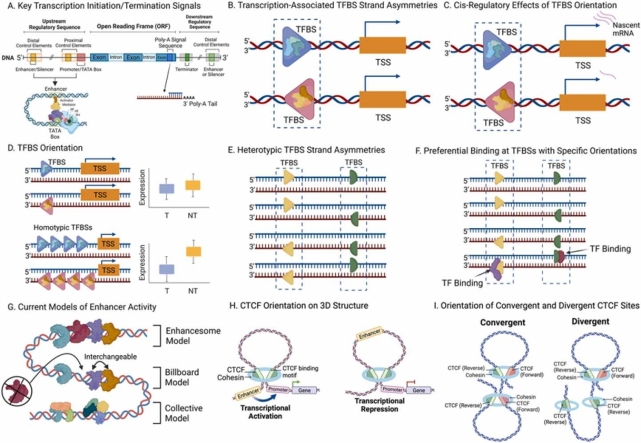

## 1 Introduction

The DNA double helix shows rotational symmetry, whereas a number of biological processes such as transcription, replication, DNA repair, and transcription factor binding have intrinsic directionalities [1], [2]. Chargaff’s first parity rule, conceived over 70 years ago, states that the number of adenines (As) equals the number of thymines (Ts), while the number of guanines (Gs) equals the number of cytosines (Cs) [3]; this parity rule can be explained by base complementarity in double-stranded DNA. Chargaff’s second parity rule states that in long genomic windows, nucleotide sequences on the two complementary strands are found with approximately the same frequency [4], [5]. Although this rule is most accurate for long nucleotide sequences [4], it holds true for most double-stranded DNA organisms, with the notable exception of certain symbiotes [6].

In contrast to Chargaff’s first parity rule, which was explained by the elucidation of the double stranded DNA structure, a comprehensive explanation for the second rule has not yet been found. Although there are no clear evolutionary advantages associated with it, the second law has been observed across diverse organisms [7]. In addition, it is not attributed to a single biological mechanism, but is likely the result of multiple genomic processes [7]. Nevertheless, some research has pointed to inversions and inverted transposition events being major contributors to the validity of this rule [8], [9], while other models have proposed stem-loop structures [10] and duplication events [11] as potential explanations.

Importantly, when investigating particular genomic localities, there are clear deviations from the second parity rule, which can be attributed to specific functional elements. Biological processes, such as transcription and replication, possess intrinsic directionality, therefore resulting in the heterogeneous distribution of information. Identification of strand asymmetries can therefore enable the detection of biological mechanisms, the identification of novel genomic elements, and the characterization of selective environmental constraints. At the same time, strand asymmetry analyses can improve computational models across biological domains, such as in the estimation of the likelihood of mutagenesis, the identification of driver mutations, in *cis*-regulatory logic, in evolution, and in disease. In this review, we provide an overview of multiple biological processes that result in the asymmetric distribution of genomic information and demonstrate the utility of strand asymmetries as a tool to decipher new biological mechanisms.

## 2 Strand asymmetries shape the nucleotide composition of diverse genomes

**Fig. 1:**
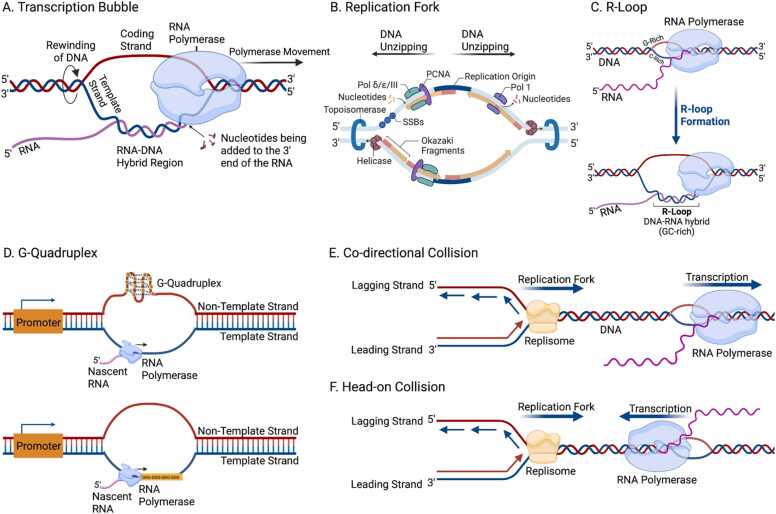
Strand asymmetries associated with replication and transcription. A. Orientation of transcription fork, displaying the template and non-template strands and the generation of a nascent RNA. B. Replication fork schematic showing continuous replication in one orientation and discontinuous replication with Okazaki fragments in the opposite strand. Topoisomerase, helicase, and polymerases are crucial enzymes in this process. C. GC-skews favor formation of secondary structures such as R-loops. D. G-quadruplex at the template strand can impede RNA polymerase movement, whereas at the non-template strand, it can facilitate transcription. E-F. Replication-transcription collisions are a cause of instability and can result in replication fork arrest, premature transcription termination and genomic instability. Types include E. co-directional collisions and F. head-on collisions (Created with BioRender.com).

In transcription, which is a directional process, the elongating RNA polymerases synthesize nascent RNA complementary to the template strand (Fig. 1a). During replication, the leading strand is replicated continuously, whereas the lagging strand is replicated in short Okazaki fragments [12], [13] (Fig. 1b). Because DNA polymerases must add nucleotide monomers in the 5' and 3' directions, a discontinuous polymerization with Okazaki fragments on the lagging orientation is necessary.

Transcriptional and replicative strand asymmetries refer to the asymmetric distribution of information such as nucleotides or motifs between the leading and lagging strands or between the template and non-template strands respectively. Both forms of asymmetry have been observed in the genomes of diverse organisms including prokaryotes, eukaryotes, and viruses [14], [15], [16], [17], [18], [19], [20], [21], [22]. These intrinsically asymmetric processes result in mutational asymmetries between the two DNA strands and have shaped the genomes of organisms across the tree of life [23]. For example, cytosine deamination occurs primarily at single-stranded DNA, resulting in C to T mutations. The likelihood of cytosine deamination is significantly higher on the leading-strand [22], and there is a higher repair rate on the lagging strand for C>T mutations [24]. As a result, in most studied bacteria, the leading strand has an excess of Gs and Ts relative to Cs and As [25]. *Borrelia burgdorferi*, a bacterium that causes Lyme disease, is one of the species with the most pronounced leading / lagging nucleotide asymmetries [26].

These asymmetries are frequently quantified with GC-skew and AT-skew, which measure statistical deviations of guanines or adenines between the two strands and which have been used to identify the location of replication origins, elucidate the direction of replication, and even to validate genome assemblies [27], [28], [29]. In the human genome, there is an enrichment of Gs and Ts relative to As and Cs on the non-template strand of genes [30]. Since the non-template DNA remains single-stranded for longer, while the template strand is used for the synthesis of the nascent RNA, cytosine deamination can explain the observed nucleotide asymmetries [31]. GC-skews favor the formation of non-canonical secondary structures including G-quadruplexes and R-loops, which are known to influence gene regulation and have also been associated with RNA polymerase pause sites in CpG island promoters [32], [33], [34], [35] (Fig. 1c-d).

In both prokaryotes and eukaryotes, a larger number of genes are usually found in the leading orientation [36], [37]. This phenomenon has been explained by a lower mutation rate, by competition between replication and gene expression [37], and as a way to limit collisions between the transcription and replication machineries [38] (Fig. 1e). A collision with the replication fork can halt transcription by the RNA polymerase in either orientation, and head-on collisions are the most common way replication is interrupted [39], [40] (Fig. 1e-f). Collisions can be a source of genomic instability, and prokaryotic genomes are therefore structured in ways that limit the number of collision events. Across 1552 studied bacterial and archaeal species, more than 90% of them subsequently display preference for their coding genes on the leading strand [41]. For instance, in the bacterium *Bacillus subtilis*, 75% of genes are transcribed in the same orientation as the direction of replication [42].

Further supporting this model, genes that are highly expressed and essential genes, such as ribosomal genes, which would experience more frequent collisions due to a higher density of elongating RNA polymerases, tend to be found on the leading strand [38], [43], [44], [45], [46]. For example, only 6% of essential genes are found on the lagging strand in *Bacillus subtilis* [47]; these essential genes found on the lagging strand in *Bacillus subtilis* have a higher rate of point mutations and non-synonymous mutations, indicating that they undergo faster adaptive evolution [48]. In addition to essential genes, longer operons are more likely to be found on the leading strand [48], [49]. As a result, head-on replication–transcription collisions result in a higher rate of mutagenesis than co-directional collisions, and there is a bias for co-orientation of transcription with replication that has been shaped by selection pressures [36]. In addition, essential genes tend to be at earlier positions in operon units in order to be more highly expressed [50], indicating how organismal genomes can be arranged to maximize protein efficacy.

In eukaryotic cells, multiple mechanisms are in place to limit collisions. These involve the separation of replication and transcription domains during S-phase [51], [52], replication fork barriers [53], coordinated changes between replication and transcription timing across different tissues or during differentiation [54], and a higher frequency of genes in early replicating domains [54], [55]. Nevertheless, replication-transcription collisions still occur in eukaryotic cells, particularly in the longer genes that require more time to be transcribed [56]. Collisions between the replication and transcription machineries are a cause of DNA damage, genomic instability, and recombination in eukaryotic cells [57].

Gene expression can be a mechanism that safeguards genome integrity. The testis is the tissue that expresses the highest number of genes in mammals; this results in a reduced mutation rate for the transcribed strand due to transcription-coupled repair, and in turn, leads to reduced population diversity across the expressed genes [58]. A study that investigated the contribution of transcriptional strand asymmetries in the usage of energetically cheaper nucleotides (“U”,”C”) in synonymous sites across 1550 prokaryotic genomes found substantial asymmetries resulting in strand-specific nucleotide usage [59]. The observed asymmetries were due to replication-related, transcriptional-related, and translational-related selection, and selection constraints were particularly amplified with higher expression levels [59].

## 3 Strand asymmetries in genes and gene features

The orientation of genes is often biased, and one extreme case of this is polycistronic gene expression, in which all genes have the same directionality. Prokaryotic operons are polycistronic, while the vast majority of eukaryotic mRNAs are monocistronic. However, it has been noted that polycistronic mRNAs can be rarely found in eukaryotic genomes [60], [61], [62]. Genes are heterogeneously distributed across the human genome. There are gene deserts, large genomic regions in which genes are largely absent, as well as gene clusters, in which gene density is significantly higher [63], [64]. This observation can be explained by common proximity-based regulation of multiple genes; genes are over-represented in early-replicating regions [65], [66].

In addition, gene pairing has been observed to be common across eukaryotic species, with genes being found in different orientations [67]. Gene pairs can be found in three orientations, which are tail to head, head to head, and tail to tail [66]. Genes in close proximity to each other are found more frequently in the head to head orientation, and this was observed for metabolism, DNA repair genes, housekeeping genes, and an unbiased set, while the expression of nearby genes has also been found to be correlated [68], [69].

Transcription in eukaryotes is inherently bidirectional, and antisense transcripts can arise from this process [70]. In contrast to mRNA transcripts, most of these antisense transcripts are unstable [71] and can be used for co-option and generation of new genes. However, it remains unclear what the exact mechanisms are that confer directional transcription. Long non-coding RNAs (lncRNAs) can be produced in the sense or antisense orientation of protein-coding genes [72]. For example, in yeast, the transcription factor Rap1 restricts transcription to the divergent orientation [73]; it remains unknown if additional transcription factors contribute to this effect.

Furthermore, key transcription initiation and termination signals, such as the TATA-box and the polyadenylation signal, display not only positional constraints but also intrinsic directionalities [74], [75], [76], and such directionalities have been used to identify genic regions [77], [78]. Nucleotide strand asymmetries have also been observed relative to splice sites [79], [80]. Strand asymmetries can be found in motifs associated with the splicing code, which are used for the recognition of core splicing signals, such as the 3’ and 5’ splice sites.

Exons and introns display opposite nucleotide strand asymmetries. In introns, Ts are more frequent than As and Gs are more abundant than Cs, a trend that is reversed in exons in both humans and mice [79]. This could serve as a mechanism to discriminate between exons and introns. Interestingly, intronless genes, in which splicing is absent, do not display these patterns [79]. Furthermore, the observed asymmetry trends do not translate to yeast. Zhang et al. found that exonic splicing enhancers and exonic splicing silencers display strand asymmetry patterns, and they utilized the observed strand asymmetry patterns to identify novel splicing regulatory elements. Another study found significant strand asymmetries in the distribution of G-quadruplexes between the template and non-template orientations relative to splice sites and provided evidence for their roles in the modulation of alternative splicing events [81]. As a result, a number of studies have used the inherent directionality in transcription initiation, splicing, and termination signals to identify mechanisms of gene regulatory control.

## 4 Mutational strand asymmetries and insights in operative biological processes

**Fig. 2:**
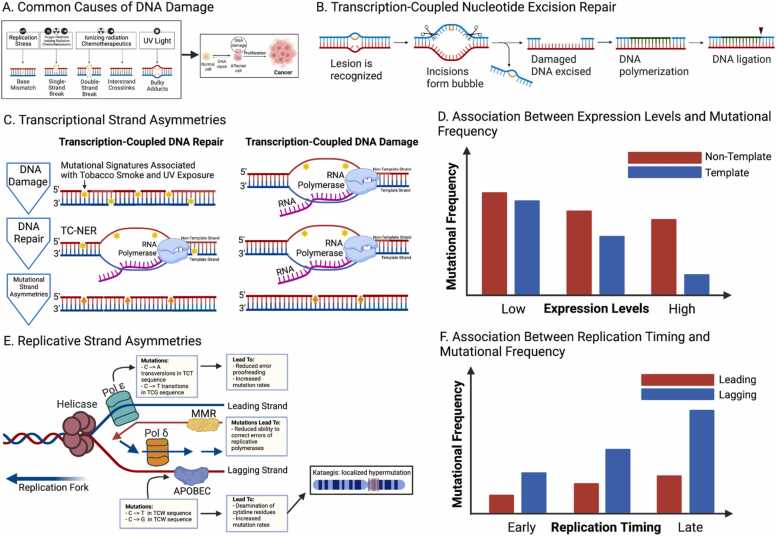
Mutational strand asymmetries. A. DNA damage from exogenous and endogenous processes can lead to the accumulation of somatic mutations and eventually can result in cancer development. B. Nucleotide excision repair schematic showing removal of transcription-blocking DNA lesions. C. In transcription-coupled repair, DNA damage at the non-template strand is more likely to escape repair from the TC-NER, leading to mutational strand asymmetries. With regards to transcription-coupled DNA damage, there is a lack of protection of the non-template strand during transcription, also leading to mutational strand asymmetry. D. Lowly expressed genes have higher mutation rates, while highly expressed genes have lower mutation rates. TC-NER activity is associated with expression levels and this results in an association between transcriptional strand asymmetry in mutations and expression levels. E. Deficiencies of proteins such as MMR or polymerase ε can lead to replicative strand asymmetries. F. Early replication timing is associated with lower mutation rates, while late replication timing is associated with higher mutation rates. For certain mutational processes, the replicative strand asymmetry aggravates with replication timing between early and late replicating regions. Schematics 2d and 2 f provide a model and do not include real data (Created with BioRender.com).

Throughout our lives, cells in the human body acquire and accumulate somatic mutations. Processes that cause the accumulation of somatic mutations can be divided into exogenous, such as UV light exposure, and endogenous, such as defects in DNA repair and oxidative damage (Fig. 2a). Therefore, mutational processes continuously shape the genome of somatic cells. Uncontrolled clonal expansion, usually through the accumulation of cancer driver mutations, can result in cancer development [82]. The vast majority of mutations in a cancer genome are passenger mutations, which have little to no effect on tumor progression. However, they can serve as signatures of operative mutational processes and also inform us about mutational strand asymmetries [83]; asymmetries can be inferred from the mutated nucleotides, depending on their frequency in leading versus lagging and template versus non-template orientations. DNA damage in either of the two complementary bases results in the same mutated site, and as a result, the base of the original DNA damage cannot be deduced with standard sequencing methods. However, substitution mutations at a reference nucleotide can be oriented on the template or the non-template strand relative to the transcriptional direction or on the leading or lagging strands relative to the directionality of the replication fork. Studies that have profiled the replicative and transcriptional strand biases relative to replication origins and transcription start sites have shown specific mutational patterns around those genomic sites [84], [85].

Strand asymmetric segregation of DNA lesions was observed in murine liver tumor genomes, resulting in chromosome-scale strand asymmetry of mutations [86]. Another study investigated how the orientation of the minor groove relative to histones influences germline and somatic mutation rate and found differences between sites with the DNA minor groove facing toward or away from the histones; this was observed across cancer types [87]. Moreover, the magnitude of the effect was higher for nucleosomes with strong rotational position, further supporting the model [87]. In a recent study, asymmetry in the distribution of structural population variants relative to the orientation of repeat elements was detected [88]. This likely reflects the jumping events of transposable elements in the population. Transposable element re-activation is frequently observed in cancer, and application of strand asymmetry analyses in structural variant datasets from cancer genomes could provide valuable mechanistic insights.

## 5 Transcriptional strand asymmetries in cancer genomes

Substitution mutations provide valuable information about underlying mutational processes. Previous research has used mutational classification of mutational signatures to further separate the standard 96 substitution classification system using the template and non-template orientation into 192 possible mutation classes [83]. The authors found strong transcriptional strand bias for mutational signatures associated with ultraviolet exposure and tobacco smoke among others [83]. In a recent study, a classification system for doublet-base substitutions and indel mutations was implemented across 4645 whole-genome and 19,184 whole-exome sequenced cancer tumors; the study identified additional mutational signatures with transcriptional strand asymmetries, which were also associated with tobacco smoke and ultraviolet exposure [89].

DNA damage is preferentially repaired at the template strand of expressed genes through transcription-coupled nucleotide excision repair (TC-NER), which removes transcription-blocking DNA lesions [90], [91] (Fig. 2b). In transcription-coupled repair, the recruitment of TC-NER correlates with expression levels, and highly transcribed genes have the most pronounced mutational strand asymmetries [92] (Fig. 2c-d). DNA damage at the non-template strand, however, is more likely to escape repair from TC-NER because it does not interfere with RNA polymerase progression and because it remains exposed as single stranded DNA, which is more likely to be mutated [93]. Therefore, transcription-associated mutations occur in part because the non-transcribed strand is single stranded and less protected from DNA damage and mutagens, which in turn can result in a higher rate of mutagenesis [94]. Recently, it was shown that transcription-associated mutagenesis is also observed in both germline and somatic mutations of higher eukaryotes at transcribed regions, a phenomenon that was previously seen primarily in microorganisms [95]. As a result, differences in DNA damage and repair between the template and non-template strands in transcribed regions are pervasive and can be reconstructed with mutational strand asymmetry analyses [96].

The accumulation of tobacco-related carcinogens at guanines in lung cancer results in the mutational imbalance of G>T site substitutions due to the preferential repair of these adducts at the template strands of expressed genes [97]. In liver cancer, a mutational signature that is correlated with alcohol consumption shows marked patterns associated with expression levels and transcription-coupled damage [98] (Fig. 2d). In bladder cancers, the mutational signature SBS92, which is enriched in smokers, has been shown to have a strong transcriptional strand asymmetry [99]. Another study oriented mononucleotide repeat tracts to observe transcriptional strand asymmetries in indel mutagenesis [100]. There is also evidence for significant differences in the strand asymmetries between introns and exons because exons are under stronger selection pressure and codon usage preference [101], [102]; there is also more efficient repair by mismatch repair (MMR) at exons [103]. However, transcription strand bias has been associated primarily with exogenous processes including tobacco smoking and UV light, which in turn are repaired by NER.

## 6 Replicative strand asymmetries in cancer genomes

Replicative strand biases are observed in cancer genomes, with one study showing significant replicative strand asymmetries across fourteen cancer types [96] (Fig. 2**e**). Systematic examination of mutational processes has indicated that replicative strand asymmetries are more common than transcriptional strand asymmetries across the mutational signatures examined [96]. In contrast with transcriptional strand asymmetries, replicative strand asymmetries are linked to endogenous processes; they are associated with repair enzyme deficiencies, such as MMR and polymerase ε deficiencies, as well as with the activity of the Apolipoprotein B mRNA editing catalytic polypeptide-like family (APOBEC) of cytidine deaminases [96]. Vöhringer et al. showed that out of twenty mutational signatures examined, nine exhibited significant replicative strand asymmetry, while only five showed significant transcriptional strand asymmetry [104]. Recently, replicative strand asymmetries have also been observed for specific mutational signatures in germline variants [105], [106].

In humans, leading and lagging strand DNA synthesis is performed primarily by polymerase ε and polymerase δ respectively [107], [108], [109]. MMR or polymerase ε deficiencies result in pronounced replicative strand asymmetries in the distribution of mutations, which indicates that these enzymes normally balance the likelihood of mutation during DNA replication [96]. It has also been observed that in certain cases, the magnitude of the replicative strand asymmetry can be associated with replication timing, with earlier replicating regions showing more pronounced replicative strand asymmetry in cancer genomes with polymerase ε deficiencies [96], [110] (Fig. 2f). Polymerase δ mutations in the exonuclease domain have also been reported; they are associated with increased mutability and show replicative strand asymmetries [111]. MMR also impacts the mutation rate between early and late replicating regions. Late replicating regions accumulate a higher number of mutations, while an MMR deficiency terminates this pattern [112]. Lujan et al. examined the contribution of MMR to replicative strand asymmetries with yeast as the model system and found that there is higher MMR efficiency for lagging-strand DNA polymerase α and DNA polymerase δ than for the leading-strand DNA polymerase ε [113]. Recent studies have also provided experimental proof for the roles of different repair enzymes in the observed mutational strand asymmetries. Zou et al. showed that the gene knockout of repair genes such as *MSH6*, *MSH2* and *MLH1* resulted in replication strand asymmetry effects in isogenic cell models [114], providing further experimental evidence regarding the contribution of the DNA mismatch repair system to mutational strand asymmetries. Knock outs of other DNA repair genes such as *EXO1* and *RNF168* showed specific transcription strand asymmetry effects [114].

Mutations associated with APOBEC, a cytidine deaminase with important roles in antiviral defense, cause off-target mutagenesis in the genome, especially at single-stranded DNA sites. There is evidence for episodic APOBEC mutagenesis across multiple cancer types [115], [116]. The APOBEC mutational signatures show a preference for early-replicating regions and highly expressed genes [117] with replicative strand asymmetry [22], [96] due to deamination of the lagging strand template during DNA replication [118]. APOBEC is also linked to kataegis, which is characterized by local strand-coordinated hypermutation [92] (Fig. 2e).

## 7 Orientation preferences in repeat elements

Transposable elements, originally discovered by McClintock [119], were initially thought of as junk DNA; however, this view has in many ways been disproven. Repeat elements represent a significant portion of the human genome and have contributed to its structure, functionalities, and evolution, while also contributing to genetic diversity between people. It is estimated that repetitive elements comprise two thirds of the human genome [120]. Some studies have suggested that transposable elements might offer an explanation for Chargaff's second parity rule [8] and account for the inversion events that could explain this rule [4], [9]. However, the integration of these elements is not random and exhibits biases in the sequence context and orientation preference [121], [122], [123], [124], as well as for preference for repeat pairs and clustering of repeat elements [125], [126], [127].

**Fig. 3:**
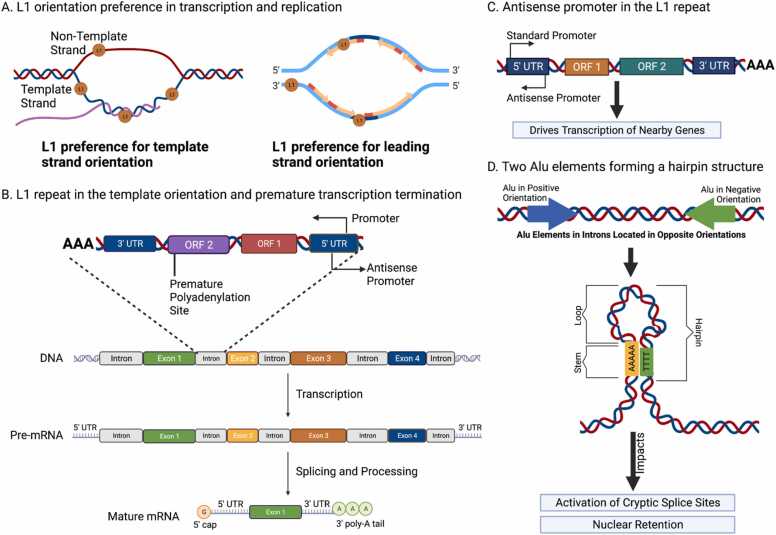
The effects of repeat element orientation. A. L1 shows an integration preference for the template strand and leading strand orientations. B. Polyadenylation signals within L1 repeats in introns in the template orientation can result in premature termination of transcription. C. Antisense promoters in L1 repeats can drive transcription of nearby genes. D. Two Alu repeats in opposite orientations can form hairpin structures (Created with BioRender.com).

In the human genome, long interspersed nuclear elements (LINE) and short interspersed nuclear elements (SINE) show significant transcriptional and replicative strand asymmetries, while long terminal repeats (LTRs) exhibit pronounced transcriptional strand asymmetries [88]. LINE-1 (L1) elements are the most abundant subclass, comprising around 17% of the human genome [128]. Only approximately 100 L1 sites are still retrotransposition competent in the germline [129] and in disease [130]. The L1 distribution in the human genome shows a preference for the leading strand orientation relative to the replication direction [124] and for the template strand orientation in transcribed regions [123], [131] (Fig. 3a). Even though there is a higher density of L1 elements at late replicating regions, integration is more likely to occur at early-replicating sites, suggesting that evolutionary selection contributes to the observed patterns in the genome. Interestingly, the smaller subset of integrations at the non-template orientation are much more likely to be pathogenic or disease-causing [132]. However, when L1 repeats are present in introns in the template orientation, they can cause premature termination of transcription due to a polyadenylation signal within the L1 element [133], [134] (Fig. 3b). On the other hand, an antisense promoter in the L1 repeat, with opposite orientation than the open reading frames of the repeat, can drive transcription of nearby genes [135] (Fig. 3**c**); this has implications for both evolution and disease.

Similarly, LTRs are more frequently found in the template orientation, and Alu repeats, which are a subset of SINE elements, also show a preference for the template orientation [136]. In lncRNAs, Alu repeats tend to be tolerated in the template strand across gene regions, whereas in the non-template strand, they tend to be found at the 3’ end [137]. Alu repeats are likely to be found clustered, closely positioned, and in direct orientation to one another [138], [139]. The orientation preference of multiple endogenous repeat elements for the template orientation in transcribed regions could be due to interference with transcription-associated signals in the non-template strand orientation, including splicing and polyadenylation motifs. Alu repeats in opposite orientations can form hairpin structures, in turn impacting biological processes such as alternative splicing and nuclear retention [125] (Fig. 3d). Overall, the orientation preference for the template strand across multiple endogenous repeat element categories could reflect the tendency to reduce the number of collisions between reverse transcription and gene transcription.

## 8 Orientation preferences in transcription factor binding

**Fig. 4:**
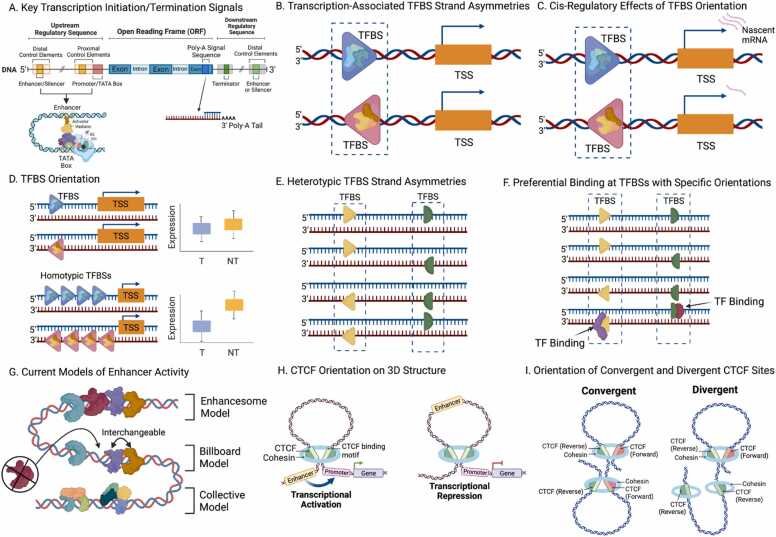
Transcription factor orientation and *cis*-regulatory grammar. A. Key transcription initiation and termination signals depicting the TATA-box and polyadenylation signal. B. and C. The orientation of a TFBS relative to the transcriptional direction in promoter upstream regions influences expression. D. and E. Orientation of TFBSs relative to each other. F. The orientation of TFBSs relative to each other influences transcription factor binding. G. Current models of enhancer activity include the enhancesome, billboard, and collective models. H. The orientation of CTCF is important for the formation of enhancer-promoter interactions and transcriptional activation. I. Convergent CTCF sites can create loops, but divergent CTCF sites disrupt the 3D structure (Created with BioRender.com).

The orientation of DNA motifs in the genome impacts diverse biological processes, including gene regulation, through its effect on co-operative transcription factor binding at cis-regulatory elements (Fig. 4a). Combinatorial transcription factor binding is instrumental in organizing gene expression patterns across developmental time points and tissues [140], [141]. Even though only a limited number of studies have thoroughly investigated the impact of TFBS orientation, there is important evidence to suggest that TFBS orientation is a major factor in gene regulatory grammar [142], [143], [144]. TFBSs can be oriented relative to transcription direction and relative to one another (Fig. 4b-c). The orientation of homotypic or heterotypic transcription factor motif pairs is biased across the genome, and their relative orientation impacts homotypic and heterotypic transcription factor complex formation [143], [144], [145], [146], [147], [148] (Fig. 4d-e).

At short inter-motif distances, the TFBS orientations impact protein-protein interactions (PPIs). In addition, even though the consensus TFBS motif of many TFs is palindromic, providing two templates for binding, there are significant binding biases depending on the orientation when considering flanking nucleotides [149]. There is also evidence to suggest that transcription factor pairs can bind to composite motifs with orientation and proximity preferences and that the composite motif sequences can differ from the constituent motif sequences of the individual transcription factors [142] (Fig. 4f). In the human transcriptome, the transcription factor binding sites for almost half of the transcription factors display strand asymmetry preference, which cannot be fully explained by nucleotide composition biases between the template and non-template strands [88]. The observed asymmetries could reflect binding preferences and not form impediments for RNA polymerase progression. Similarly, both at promoter upstream and downstream regions, there is orientation bias for a number of transcription factors [88]. In plants, orientation preference of TFBSs has been observed close to the transcription start site, which was attributed to background strand asymmetries in the dinucleotide composition of promoter upstream regions [150]. An association with expression levels was not identified.

At the core promoter, a number of motifs are positioned with respect to orientation, distance, and order preferences. For instance, transcription initiation in TATA-box-containing promoters requires the orientation and correct positioning of promoter-related motifs, including the initiation element, the TATA-box, and the upstream and downstream promoter elements, among others [74], [75]. Reversal of the TATA-box orientation can significantly reduce transcription levels [151]. In promoters with TATA and Inr motifs, correct spacing and orientation are important constituents for a synergistic effect [152]. At the 5’ end of the first intron in the non-template strand, G-quadruplexes and GrIn1 motifs have been shown to be associated with promoter-proximal pausing [153].

With regards to enhancers, studies that have investigated their mechanism of function have led to the proposition of two models, and there is currently evidence to support both of them. The “enhanceosome model” states that the function of the enhancer is dependent on the orientation, positioning, and order of TF binding sites, with changes in them resulting in significant changes in the enhancer’s activity [154] (Fig. 4g). The interferon-beta (IFN-beta) enhanceosome, which is highly conserved and for which an atomic model of cooperative TF binding has been produced, provided the first evidence to support the enhanceosome model [155], [156]. For example, within the IFN-beta enhanceosome, the ATF-2–c-jun heterodimer binds in a specific orientation which is necessary for the formation of the complex between ATF-2–c-jun and interferon regulatory factor 3 [157].

Second, the “billboard model”, which is also referred to as the information display model, proposes a more flexible structure for enhancer grammar in which the combination, orientation, order, and distance of cognate motifs are not fixed, but can instead vary without impacting enhancer function [157], [158] (Fig. 4g). In this model, only the binding sites themselves are critical. A number of studies have provided support for the billboard model [159], [160], indicating that both the enhanceosome and billboard models are likely to be true dependent on the specific enhancer.

Multiple studies have provided experimental evidence for the effect of orientation and spacing in *cis*-regulation. In a breakthrough study, researchers performed consecutive affinity-purification systematic evolution of ligands by exponential enrichment (CAP-SELEX) experiments, with which they examined 9400 TF–TF–DNA interactions. Interestingly, they were able to show that both the orientation and distance between the TF motifs determined heterodimer formation for a plethora of TF pairs [142]. Using massively parallel reporter assays (MRPAs), the orientation of enhancer tiles was found to have limited effects on expression levels [161]. However, this study did not capture orientation differences of individual TFs or of TF pairs within the enhancer tiles.

The transcription factor Yin-Yang can act as an activator or a repressor depending on motif orientation and positioning [162]. The orientation of the nuclear receptor for 1,25-dihydroxyvitamin D3 response elements in the basal promoter of the human calbindin D9k gene and the rat osteocalcin gene can change the expression 10-fold, and therefore, the orientation of the response elements dramatically influences the transcriptional response [163]. GABP–CREB1 motifs tend to be spaced with a one or two base pair gap with the two motifs in opposite orientations [164]. In the case of AP-1 transcription factor, the motif orientation, as well as its flanking base pairs at AP-1 binding sites, influence homo- and hetero-dimerization, and heterodimers of Fos and Jun bind in a preferred orientation [149], [165], [166]. In the IFN-β enhanceosome, the ATF-2–c-jun heterodimer does not show an orientation preference in the absence of IRF-1, whereas in its presence, it adopts an orientation-specific binding [157]. Therefore, in this particular case, the sequence orientation and the presence of specific proteins dictates the orientation of heterodimeric transcription factor binding. Another example of orientation preference has been observed in the NF-κB p50-p65 heterodimer, which is controlled by half-sites in the κB motif [167], [168].

The positioning of TFBSs within a nucleosome influences transcription factor binding, which can subsequently stabilize or destabilize a nucleosome [169], [170]. TFBSs can be found at different positions, such as near the edge or center of the nucleosome. Furthermore, studies have shown that TFs display directional binding to nucleosomes. TFBSs positioned along a nucleosome’s surface can face inward or outward. For the TFBSs of many transcription factors, especially of ETS and CREB bZIP factors, there is a preference for the end of the nucleosomal DNA or for periodic positions on the solvent-exposed side of the DNA [171]. This is likely due to steric hindrance and scaffolding by the nucleosome, resulting in specific positioning and orientation of TFBSs [171]. Furthermore, DNase I hypersensitivity analysis followed by sequencing (DNase-seq) experiments revealed unidirectional opening of chromatin relative to pioneer transcription factor motifs, with four out of the eight pioneer transcription factor families opening chromatin in a single orientation [172]. Nucleosome oriented binding has been observed for multiple pioneer transcription factors, including GATA3 and FOXA1 [173]; these TFs are able to bind to closed chromatin, recruit nucleosome remodelers, histone modification enzymes, and other transcription factors upon binding, and change the accessibility of a cis-regulatory region. However, additional research is required to examine the interplay between chromatin structure and the orientation of TFBSs and TF complexes.

## 9 CTCF motif orientation and genome organization

One of the most notable examples has been the CCCTC-binding factor (CTCF), which contributes to the formation of topologically-associating domains (TADs). Enhancer-promoter interactions are constrained within TADs, with the orientation of CTCF sites being important for their formation (Fig. 4h). The vast majority of CTCF sites are found to be bound by cohesin [174], which is associated with transcription factors and present in almost all active enhancer regions [175]. CTCF and the cohesin complex colocalize on chromatin, and their organization can help regulate three-dimensional genome structure through chromatin loop formation [176], [177]. These protein-mediated loops bring two loci that lie far apart along the chromosome into closer physical proximity; the CTCF binding sites stop loop extrusion with the ring-like cohesin complex [178]. The process of loop extrusion has been shown to link promoters and enhancers, be correlated with gene activation, and be conserved across both cell types and species [177], [179] (Fig. 4i). Interestingly, Rao et al. demonstrated that the deletion of CTCF sites interferes with loop formation and that after cohesin loss, loop domains disappear [177]. On the other hand, during cohesin recovery, the loop domains form again in minutes [177].

Loop extrusion can increase contact between loci that would typically lie in different sub-compartments [177]. The genome is separated into intervals based on distinctive histone marks, and these intervals are assigned to two compartments, A or B [177]. Intervals of the same type demonstrate increased contact frequency with one another, and loci in a compartment often form contact domains. When cohesin is lost, compartmentalization is preserved, demonstrating that it does not rely on cohesin, unlike the loop extrusion mechanism [177]. The loop extrusion mechanism interferes with compartmentalization by promoting the co-localization of loci not necessarily from the same compartment [177]. These loops are predominantly formed (greater than 90%) by convergent CTCF motif pairs that are asymmetric and face each other [180]. When their orientation is reversed, the 3D structure is disrupted (Fig. 4i).

Disruption of the loop extrusion mechanism has been associated with cancer due to alterations in enhancer-gene interactions [178]. This disruption is a result of the hypermutation of CTCF/cohesin binding sites, which are functional and alter CTCF binding, in almost all cancer types [175], [181]. Skin cancers specifically demonstrate distinct asymmetric mutations at CTCF-cohesin binding sites that form independently of replication timing; the specific mutations can be attributed to UV radiation and uneven nucleotide excision repair [181]. This mutation bias points towards cohesin being important for stabilization during CTCF-DNA binding and for impairing NER [181].

## 10 Conclusions

In this review, we have highlighted a number of genomic processes that are associated with strand asymmetries and have presented many of the underlying mechanisms that contribute to the asymmetric distribution of genomic features in organismal genomes. Strand asymmetries shape the nucleotide composition of viral, prokaryotic, and eukaryotic genomes and are genomic signatures of the biological processes that shape them. We have also highlighted the contribution of strand asymmetries in gene regulation, splicing, transcription factor binding, and retrotransposition. In addition, we summarize evidence regarding how mutational strand asymmetries reveal insights into DNA damage and repair in human health and disease. We argue that the implementation of sensitive methods to detect strand asymmetries in biological problems will enable breakthroughs in our understanding of genome biology.

The directionality of information in the DNA molecule is reflected in the orientation of motifs, genes, and other genomic elements. To conclude, an analogy can be drawn between genomic strand asymmetries and the road code, which dictates the rules by which vehicles have to move around in cities and with traffic signs that give instructions to road users. Similar to that, the orientation of motifs, genes, and other genomic elements in the genome provides instructions on how they should be interpreted.

## CRediT authorship contribution statement

I.G.S. conceived and supervised the study. C.M., A.Z. and I.G.S. wrote the manuscript. All authors approved the final manuscript as submitted and agree to be accountable for all aspects of the work.
